# Dand

**DOI:** 10.4103/0970-0358.53026

**Published:** 2009

**Authors:** Suhas V. Abhyankar, Ananta Kulkarni, Naveen K. Agarwal

**Affiliations:** Department of Plastic Surgery, Padmashree Dr. D Y Patil Hospital and Research Centre, Sector-5, Nerul, Navi Mumbai, India

**Figure 1:**
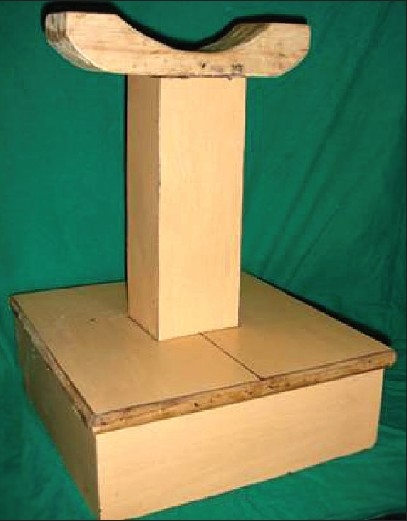
T-shaped wooden device

**Figure 2:**
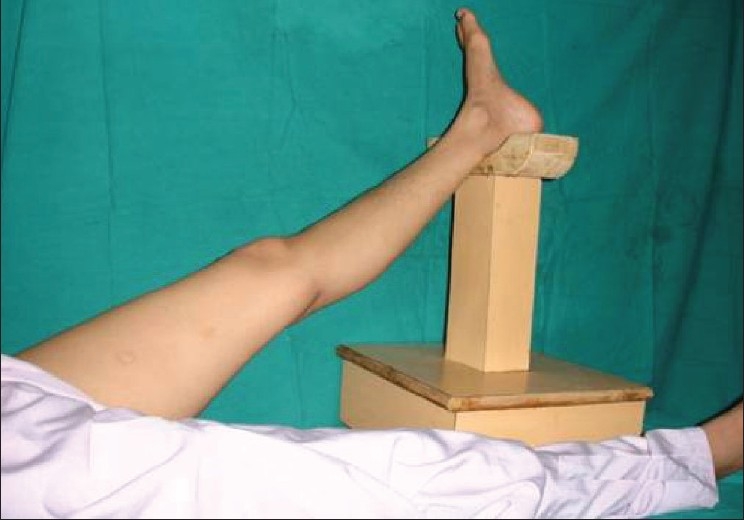
Device used Intraoperatively

**Figure 3:**
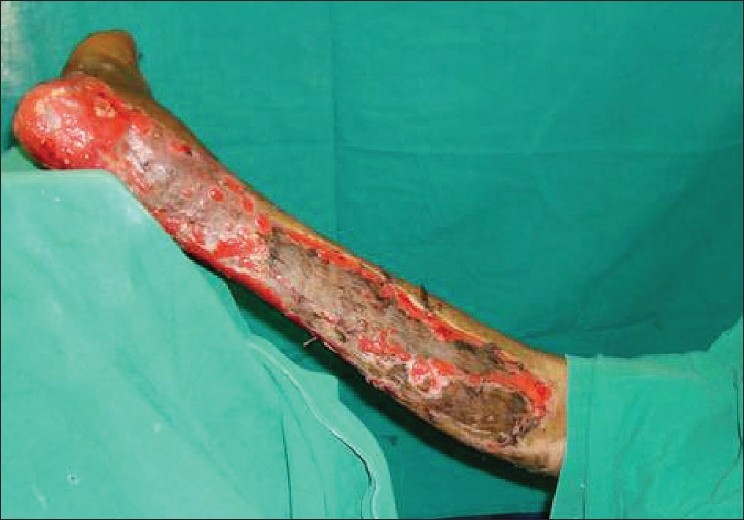
Intraoperative image after draping

Sir,

In the majority of reconstructive procedures skin graft is often required to cover the defect and usually skin grafts are harvested from the thigh region. Whether it is an Institute or nursing home setup, manpower is required to hold the inferior extremity during the preparation of the donor area. It becomes exhausting for the assistant to hold the limb if the patient is hefty. This can result in muscle fatigue, and waste of manpower. To overcome this problem, to save time and to reduce manpower, the authors have made a simple device for this purpose [Figures 1–3].

This simple device is made of teak-wood, which has a transverse limb, with a saucer shaped platform in the middle; this transverse limb has a vertical support also made of teak-wood and this assembly rests on a square platform which is made of plywood, so that the foot rests on the transverse limb of the device.

When using this device there is no need to hold the inferior extremity, thus saving manpower and thereby preventing muscle fatigue in the assistant.

It even allows the operating surgeon to operate on the lower limb or foot without applying tourniquet since limb elevation decreases blood supply, facilitates venous drainage, thereby reducing bleeding during the surgery. Moreover, this platform can be easily covered using a sterile drape.

Any carpenter can make this platform, which is cost-effective and simple to make.

**Figure 4:**
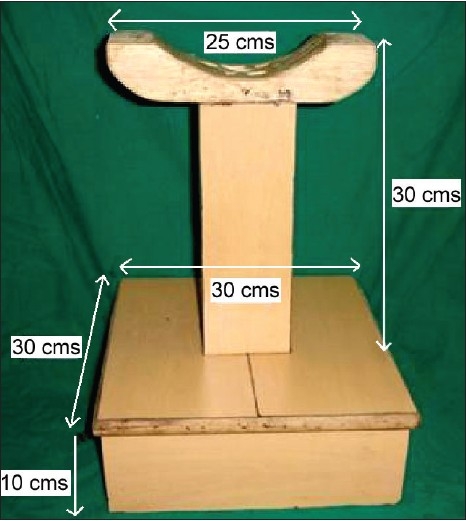
Dimensions

Dimension of the device [Figure 4] is as follows:

- Height - 30 cm
- Width - 25 cm
- Plywood box - 30 cm × 30 cm × 10 cm.

This particular design was taken from the T-shaped wooden device which was used since ancient times in India by saints to rest their arm while chanting scriptures. In Sanskrit this wooden device has been referred to as “Dand”.

This is an application of a simple idea into day to day practice and the authors feel that most of plastic surgeons must be experiencing the same difficulty while preparing the lower limb for graft harvesting. The authors have been using this device since the last two years. For many surgeries on foot and leg Dand is used without the requirement of tourniquet.

